# Mixed-metal phosphates K_1.64_Na_0.36_TiFe(PO_4_)_3_ and K_0.97_Na_1.03_Ti_1.26_Fe_0.74_(PO_4_)_3_ with a langbeinite framework

**DOI:** 10.1107/S2056989021011877

**Published:** 2021-11-16

**Authors:** Igor V. Zatovsky, Nataliia Yu. Strutynska, Ivan V. Ogorodnyk, Vyacheslav N. Baumer, Nickolai S. Slobodyanik, Denis S. Butenko

**Affiliations:** a F.D. Ovcharenko Institute of Biocolloidal Chemistry, NAS Ukraine, 42 Acad. Vernadskoho blv., 03142 Kyiv, Ukraine; b Department of Inorganic Chemistry, Taras Shevchenko National University of Kyiv, 64/13, Volodymyrska Str., 01601 Kyiv, Ukraine; c ShimUkraine LLC 18, Chigorina Str., office 429, 01042 Kyiv, Ukraine; d STC "Institute for Single Crystals", NAS of Ukraine, 60 Lenin ave., 61001 Kharkiv, Ukraine; e Shenzhen Key Laboratory of Solid State Batteries, Academy for Advanced Interdisciplinary Studies, Southern University of Science and Technology, Shenzhen 518055, People’s Republic of China; f Guangdong Provincial Key Laboratory of Energy Materials for Electric Power, Southern University of Science and Technology, Shenzhen 518055, People’s Republic of China

**Keywords:** crystal structure, phosphate, mixed occupancy, framework structure

## Abstract

Single crystals of the langbeinite-type phosphates K_1.65_Na_0.35_TiFe(PO_4_)_3_ and K_0.97_Na_1.03_Ti_1.26_Fe_0.74_(PO_4_)_3_ were grown by crystallization from high-temperature self-fluxes in the system Na_2_O–K_2_O–P_2_O_5_–TiO_2_–Fe_2_O_3_ using fixed molar ratios of (Na+K):P = 1.0, Ti:P = 0.20 and Na:K = 1.0 or 2.0 over the temperature range 1273–953 K. The three-dimensional framework of the two isotypic phosphates are built up from [(Ti/Fe)_2_(PO_4_)_3_] structure units containing two mixed [(Ti/Fe)O_6_] octa­hedra (site symmetry 3) connected *via* three bridging PO_4_ tetra­hedra. The potassium and sodium cations share two different sites in the structure that are located in the cavities of the framework. One of these sites has nine and the other twelve surrounding O atoms.

## Abstract 2

K_1.65_Na_0.35_TiFe(PO_4_)_3_ and K_0.97_Na_1.03_Ti_1.26_Fe_0.74_(PO_4_)_3_ are isotypic and crystallize in the langbeinite structure type. K^+^ and Na^+^ cations, and Ti^3+^, Ti^4+^ and Fe^3+^ cations, respectively, share the same sites in the crystal structure.

## Chemical context

Over the last decade, numerous research efforts have been directed towards the creation of new phosphate materials for Li- or Na-ion batteries (Nose *et al.*, 2013 ▸; Zhang *et al.*, 2021 ▸). In particular, significant progress has been made for complex phosphates with general formula *M* ^I^_1+_ *_x_Z*_2_(PO_4_)_3_ (*M* ^I^ = Li, Na; *Z* = polyvalent metals; *x* values can range from 0 to 3; Zatovsky *et al.*, 2016 ▸) adopting NASICON-type structures. The composition of phosphates with a langbeinite-type structure is very similar to the composition of NASICON-type ones, and langbeinite-type phosphates are also considered to be potential hosts for new electrode materials (Luo *et al.*, 2019 ▸). However, langbeinite-type phosphates with a composition *M* ^I^_1+_ *_x_Z*_2_(PO_4_)_3_ (*x* = 0–1) can only be prepared with large monovalent cations (*e.g.*, K, Rb, Cs, NH_4_; Norberg, 2002 ▸; Ogorodnyk *et al.*, 2007*a* ▸). The langbeinite-type structure has only been reported for Na_2_ *Z* ^III^Ti(PO_4_)_3_ (*Z* ^III^ = Cr, Fe; Isasi & Daidouh, 2000 ▸). More recently, a good prospect for using such kinds of materials as anodes for Na-ion batteries has been predicted because of the recently reported migration mechanisms in langbeinite-type Na_2_CrTi(PO_4_)_3_ determined by atomic simulation (Luo *et al.*, 2019 ▸). However, according to Wang *et al.* (2019 ▸), the phosphate Na_2_CrTi(PO_4_)_3_ belongs to the family of compounds with a NASICON-type structure. Therefore, the issue of preparing Na-containing langbeinite-type phosphates requires further research and development. In recent years, the synthesis of K/Na-containing complex phosphates has been realized using the self-flux method and resulted in the compounds K_1.75_Na_0.25_Ti_2_(PO_4_)_3_ (Zatovsky *et al.*, 2018 ▸) and K_0.877_Na_0.48_Ti_2_(PO_4_)_3_ (Strutynska *et al.*, 2016 ▸).

Here, we report the preparation, structure analysis and characterization of two new mixed-metal phosphates K_1.64_Na_0.36_TiFe(PO_4_)_3_ (I) and K_0.97_Na_1.03_Ti_1.26_Fe_0.74_(PO_4_)_3_ (II), which are isotypic with the mineral langbeinite, K_2_Mg_2_(SO_4_)_3_ (Zemann & Zemann, 1957 ▸; Mereiter, 1979 ▸).

## Structural commentary

**Figure 1:**
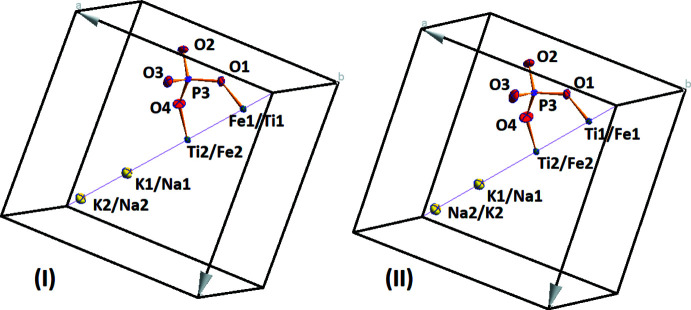
A view of the asymmetric units of (I) and (II), with displacement ellipsoids drawn at the 50% probability level.

**Figure 2:**
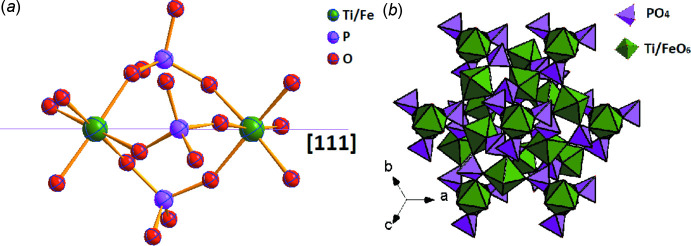
(*a*) [(Ti/Fe)_2_(PO_4_)_3_] building unit and (*b*) three-dimensional framework for (I) and (II).

As it is illustrated in Fig. 1 ▸, two pairs of mixed sites occupied by alkali metals (K/Na) and transition metals (Ti/Fe) are located on threefold rotation axes (Wyckoff position 4 *a*), whereas the P and all O atoms occupy general sites (12 *b*). In the structures, the main structural element for building of the three-dimensional framework is a [(Ti/Fe)_2_(PO_4_)_3_] fragment consisting of two mixed-metal [(Ti/Fe)O_6_] octa­hedra and three PO_4_ tetra­hedra (Fig. 2 ▸ *a*). Such building units run in three orthogonal directions along the cubic space diagonals (Fig. 2 ▸ *b*), which is typical for the langbeinite-related family of compounds (sulfates, phosphates, vanadates *etc*, Ogorodnyk *et al.*, 2007*a* ▸).

Two octa­hedrally coordinated sites (Ti1/Fe1) and (Ti2/Fe2) show mixed occupancy with an Fe:Ti ratio close to 1:1. For (I), the Ti occupancy is 0.48 (3) for the *M*1 site, while for the *M*2 site it is 0.52 (3); for (II), the Ti occupancy is 0.61 (2) for the *M*1 site and 0.65 (2) for the *M*2 site. In the case of (I), this corresponds to Fe^3+^ and Ti^4+^ cations, while for (II), the simultaneous presence of Fe^3+^, Ti^3+^ and Ti^4+^ is suggested. The prepared crystals of (II) are violet in color and the Ti^3+^:Ti^4+^ ratio is about 1:4 taking into account the total charge of the cationic part of the compound. Partial self-reduction of Ti^4+^ to Ti^3+^ often accompanies the synthesis of langbeinite-type complex phosphates in fluxes of multicomponent systems when various trivalent or divalent metals are present (Gustafsson *et al.*, 2005 ▸; Zatovskii *et al.*, 2006 ▸). For structures (I) and (II), the [Ti/FeO_6_] octa­hedra are slightly distorted (Tables 1 ▸ and 2 ▸). The range of *M*—O bond lengths [1.938 (2) – 1.976 (3) Å] is close to those in other langbeinite-related phosphates containing Ti and transition metals, such as K_2_Fe_0.5_Ti_1.5_(PO_4_)_3_ [1.940 (2)–1.992 (2) Å]; K_2_Ni_0.5_Ti_1.5_(PO_4_)_3_ [1.938 (5)–1.962 (5) Å]; K_2_Co_0.5_Ti_1.5_(PO_4_)_3_ [1.945 (2)–1.974 (2) Å]; K_2_Mn_0.5_Ti_1.5_(PO_4_)_3_ [1.961 (2)–2.002 (2) Å] (Ogorodnyk *et al.*, 2008 ▸, 2007*b* ▸, 2006 ▸). The P—O distances for both structures are in the narrow ranges of 1.516 (4)–1.523 (3) for (I) and 1.517 (3)–1.523 (2) Å for (II).

There are two sites where the alkali metal cations reside (Fig. 1 ▸). The first one, (K/Na)1 is occupied by K^+^ and Na^+^ cations at ratios of 0.85 (2):0.15 (2) and 0.676 (18):0.324 (18) for (I) and (II), respectively. The [(K/Na)1O_9_] polyhedra show three sets of (K/Na)—O distances assuming a cut-off value for the contact lengths of 3.129 (4) Å; the bond lengths are similar for both structures (Tables 1 ▸ and 2 ▸). The coordination environment of the alkali cations related to the (K/Na)2 site consists of twelve O-atom neighbours with (K/Na)2—O distances ranging from 2.843 (3) to 3.237 (3) Å, which includes four sets of distances (Tables 1 ▸ and 2 ▸). For this site, the K:Na ratios are 0.80 (3):0.20 (3) for (I) and 0.294 (19):0.706 (19) for (II). An inter­esting fact is that the substitution of potassium by sodium in the position (K/Na)2 is greater for (II) than for (I), but the (K/Na)2—O distances change insignificantly.

## Synthesis and crystallization

Phosphates (I) and (II) were obtained from the melts of the system Na_2_O–K_2_O–P_2_O_5_–TiO_2_–Fe_2_O_3_ at fixed molar ratios of (Na+K)/P = 1.0, Ti/P = 0.20 and different values of Na/K = 1.0 or 2.0 over the temperature range 1273–953 K. All initial components *M^I^* H_2_PO_4_ (*M^I^* = Na, K), Fe_2_O_3_ and TiO_2_ were of an analytical grade.

A mixture of KH_2_PO_4_ (10 g), NaH_2_PO_4_ (8.82 g), Fe_2_O_3_ (2.35 g) and TiO_2_ (2.35 g) was used for the preparation of (I), while a mixture of KH_2_PO_4_ (10 g), NaH_2_PO_4_ (17.64 g), Fe_2_O_3_ (3.53 g) and TiO_2_ (3.53 g) was used for the preparation of (II). In both cases, the mixtures of calculated amounts of starting components were ground in an agate mortar and melted in a platinum crucible at 1273 K. The obtained melts were kept under isothermal conditions for 2 h for dissolving of the corresponding TiO_2_ + Fe_2_O_3_ mixture in the phosphate melt. Then the temperature was decreased with a rate of 25 K h^−1^ to 953 K and kept at this temperature for 2 h before cooling down to room temperature by turning off the furnace power. The obtained crystalline phases were separated from soluble salts by leaching with hot water and dried at 373 K.

**Figure 3:**
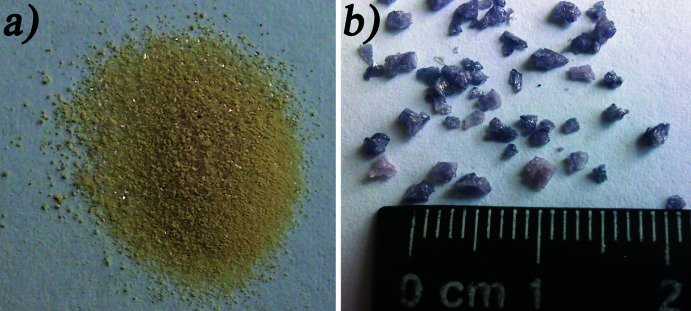
Photographs of single crystals of (*a*) (I) and (*b*) (II).

The molar ratio Na/K in the initial melts had an influence on the composition of the obtained crystals. Light-yellow crystals formed in the melt with a ratio of Na:K = 1.0 while violet crystals were obtained for a ratio Na:K = 2.0 (Fig. 3 ▸). It should be noted that increasing the amount of sodium in the initial melts to a ratio Na/K = 2.0 caused the growth of crystals with sizes of 2–3 mm (Fig. 3 ▸ *b*) in length.

The chemical compositions of the prepared samples (qu­anti­tative determination of K, Na, Ti, Fe and P) were confirmed by ICP–AES with a Shimadzu ICPE-9820 spectrometer. The analyses showed that the molar ratios of K:Na:Ti:Fe:P were close to 1.65:0.35:1:1:3 for (I) and 1:1:1.25:0.75:3 for (II).

**Figure 4:**
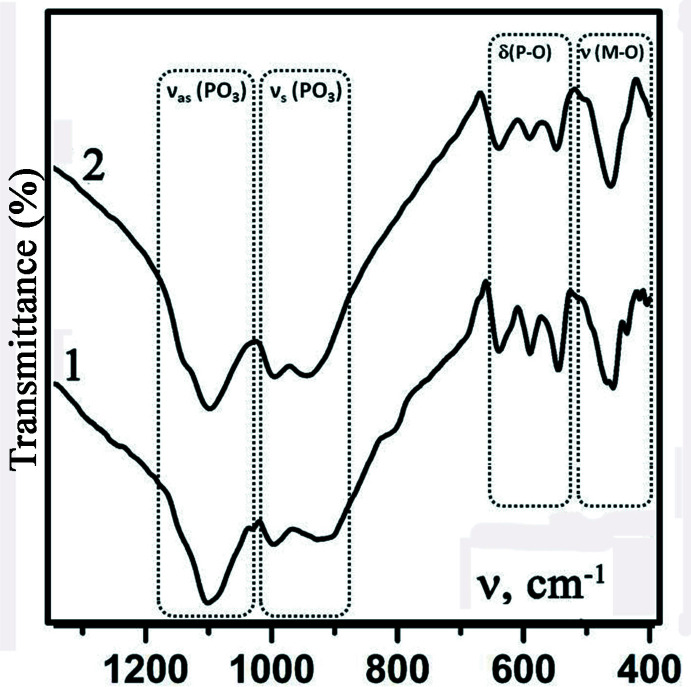
FTIR spectra of (I) (curve 1) and (II) (curve 2).

The phosphates (I) and (II) were further characterized using Fourier transform infrared (FTIR) spectroscopy. The spectra were obtained using a PerkinElmer Spectrum BX spectrometer in the range 4000–400 cm^−1^ (at 4 cm^−1^ resolution) with sample material pressed into KBr pellets. The FTIR spectra for both compounds are similar in band positions of vibration modes (Fig. 4 ▸). The broad and intense bands in the frequency region 1150–900 cm^−1^ are characteristic for P—O stretching vibrations [*ν*_as_(PO_3_) – region 1150–1090 cm^−1^ and *ν*_s_(PO_3_) – region 1020–900 cm^−1^] of the PO_4_ tetra­hedron. The band group at 650–550 cm^−1^ is caused by bending *δ*(P—O) vibrations of P—O bonds. Some differences in the spectra were observed in the range 500–400 cm^−1^, which are due to *X*—O (*X* = Ti, Fe) vibrations and correlate with insignificant differences in the composition of the prepared compounds (I) and (II).

## Refinement

Crystal data, data collection and structure refinement details are summarized in Table 3 ▸. According to the results of the chemical analysis, large qu­anti­ties of Na and Ti are present in the structures. Taking into account possible coordination spheres of Na and Ti and previously reported langbeinite-type phosphates with a mixed-metal framework, we supposed that Ti occupies the same sites as Fe, and Na the same positions as K. Hence, the corresponding positions of Fe1 and Fe2, K1 and K2 were occupied with Ti and Na, respectively. As the Fe(Ti) positions are part of the rigid framework, we assumed that these sites show full occupancy, while the sites related with the alkali metal can be fully or partially occupied. At a first approach, the occupancies were refined using linear combinations of free variables (SUMP restraint). Two SUMP restraints were applied to occupancies of Fe1(Ti1) and Fe2(Ti2) sites. One more SUMP restraint was then applied to the sum of valence units of all metal-atom positions. This refinement resulted in satisfactory reliability factors. It was found that the occupancies of K1(Na1) and K2(Na2) are close to 1. Thus, to simplify the refinement we tried to refine the occupancies with free variable constraints instead of SUMP restraints while keeping the alkali metal site occupancies equal to 1. To each refined position, a unique free variable constraint was applied, plus constrained identical coordinates and ADPs for each site. The resulting reliability factors were found to be almost equal to those where the SUMP restraints were used. For the final refinement cycles, the second approach was applied to both structures.

## Supplementary Material

Crystal structure: contains datablock(s) global, I, II. DOI: 10.1107/S2056989021011877/wm5625sup1.cif

Structure factors: contains datablock(s) I. DOI: 10.1107/S2056989021011877/wm5625Isup2.hkl

Structure factors: contains datablock(s) II. DOI: 10.1107/S2056989021011877/wm5625IIsup3.hkl

CCDC references: 2121192, 2121193

Additional supporting information:  crystallographic information; 3D view; checkCIF report

## Figures and Tables

**Table 1:** Selected bond lengths (Å) for (I)

Fe1—O2^i^	1.954 (3)	K2—O2^vi^	2.911 (4)
Fe1—O1	1.976 (3)	K2—O4^vii^	3.007 (4)
Fe2—O3^ii^	1.938 (3)	K2—O4^viii^	3.231 (4)
Fe2—O4^iii^	1.970 (3)	P3—O4	1.516 (4)
K1—O1^iv^	2.830 (4)	P3—O2	1.522 (3)
K1—O2^v^	3.019 (4)	P3—O3	1.523 (3)
K1—O4^v^	3.129 (4)	P3—O1	1.523 (3)
K2—O3^v^	2.854 (4)		

Symmetry codes: (i) -z, x-{\script{1\over 2}}, -y+{\script{1\over 2}}; (ii) y+{\script{1\over 2}}, -z+{\script{1\over 2}}, -x+1; (iii) z, x, y; (iv) -z+{\script{1\over 2}}, -x+1, y+{\script{1\over 2}}; (v) z+{\script{1\over 2}}, -x+{\script{3\over 2}}, -y+1; (vi) x+{\script{1\over 2}}, -y+{\script{3\over 2}}, -z+1; (vii) -y+1, z+{\script{1\over 2}}, -x+{\script{3\over 2}}; (viii) -z+1, x+{\script{1\over 2}}, -y+{\script{3\over 2}}.

**Table 2:** Selected bond lengths (Å) for (II)

Fe1—O2^i^	1.940 (2)	K2—O2^vi^	2.910 (3)
Fe1—O1	1.974 (2)	K2—O4^v^	2.982 (4)
Fe2—O3^ii^	1.938 (2)	K2—O4^vii^	3.237 (3)
Fe2—O4	1.954 (2)	P3—O4	1.517 (3)
K1—O1^iii^	2.820 (3)	P3—O3	1.518 (2)
K1—O2^iv^	3.009 (3)	P3—O2	1.520 (2)
K1—O4^v^	3.122 (3)	P3—O1	1.523 (2)
K2—O3^v^	2.843 (3)		

Symmetry codes: (i) -z, x-{\script{1\over 2}}, -y+{\script{1\over 2}}; (ii) -x+1, y+{\script{1\over 2}}, -z+{\script{1\over 2}}; (iii) -z+{\script{1\over 2}}, -x+1, y+{\script{1\over 2}}; (iv) -y+1, z+{\script{1\over 2}}, -x+{\script{3\over 2}}; (v) z+{\script{1\over 2}}, -x+{\script{3\over 2}}, -y+1; (vi) -y+{\script{3\over 2}}, -z+1, x+{\script{1\over 2}}; (vii) -z+1, x+{\script{1\over 2}}, -y+{\script{3\over 2}}.

**Table 3:** Experimental details

	(I)	(II)
Crystal data		
Chemical formula	K_1.65_Na_0.35_TiFe(PO_4_)_3_	K_0.97_Na_1.03_Ti_1.26_Fe_0.74_(PO_4_)_3_
*M*_r_	461.19	448.16
Crystal system, space group	Cubic, *P*2_1_3	Cubic, *P*2_1_3
Temperature (K)	293	293
*a* (Å)	9.82010 (13)	9.7945 (1)
*V* (Å^3^)	947.00 (4)	939.61 (3)
*Z*	4	4
Radiation type	Mo *K*α	Mo *K*α
μ (mm^−1^)	3.69	3.27
Crystal size (mm)	0.13 × 0.10 × 0.07	0.15 × 0.11 × 0.08

Data collection		
Diffractometer	Oxford Diffraction Xcalibur-3	Oxford Diffraction Xcalibur-3
Absorption correction	Multi-scan (Blessing, 1995 ▸)	Multi-scan (Blessing, 1995 ▸)
*T*_min_, *T*_max_	0.675, 0.782	0.622, 0.835
No. of measured, independent and observed [*I* > 2σ(*I*)] reflections	1897, 847, 829	10546, 837, 833
*R*_int_	0.027	0.026
(sin θ/λ)_max_ (Å^−1^)	0.681	0.681

Refinement		
*R*[*F* ^2^ > 2σ(*F* ^2^)], *wR*(*F* ^2^), *S*	0.025, 0.064, 1.14	0.016, 0.043, 1.12
No. of reflections	847	837
No. of parameters	63	63
Δρ_max_, Δρ_min_ (e Å^−3^)	0.48, −0.37	0.29, −0.27
Absolute structure	Flack *x* determined using 339 quotients [(*I* ^+^)−(*I* ^−^)]/[(*I* ^+^)+(*I* ^−^)] (Parsons *et al.*, 2013 ▸)	Flack *x* determined using 349 quotients [(*I* ^+^)−(*I* ^−^)]/[(*I* ^+^)+(*I* ^−^)] (Parsons *et al.*, 2013 ▸)
Absolute structure parameter	−0.02 (2)	−0.042 (11)

Computer programs: *CrysAlis CCD* (Oxford Diffraction, 2006 ▸), *CrysAlis RED* (Oxford Diffraction, 2006 ▸), *SHELXS* (Sheldrick, 2008 ▸), *SHELXL* (Sheldrick, 2015 ▸), *DIAMOND* (Brandenburg, 2006 ▸), *WinGX* (Farrugia, 2012 ▸), *enCIFer* (Allen *et al.*, 2004 ▸) and *publCIF* (Westrip, 2010 ▸).

## supplementary crystallographic information

### Potassium sodium titanium iron tris(phosphate) (I) Crystal data

**Table:**

K_1.65_Na_0.35_TiFe(PO_4_)_3_	*D*_x_ = 3.235 Mg m^−^^3^
*M**_r_* = 461.19	Mo *K*α radiation, λ = 0.71073 Å
Cubic, *P*2_1_3	Cell parameters from 1897 reflections
Hall symbol: P 2ac 2ab 3	θ = 2.9–29.0°
*a* = 9.82010 (13) Å	µ = 3.69 mm^−^^1^
*V* = 947.00 (4) Å^3^	*T* = 293 K
*Z* = 4	Tetrahedron, light yellow
*F*(000) = 896.8	0.13 × 0.10 × 0.07 mm

### Potassium sodium titanium iron tris(phosphate) (I) Data collection

**Table:**

Oxford Diffraction Xcalibur-3 diffractometer	829 reflections with *I* > 2σ(*I*)
Graphite monochromator	*R*_int_ = 0.027
φ and ω scans	θ_max_ = 29.0°, θ_min_ = 2.9°
Absorption correction: multi-scan (Blessing, 1995)	*h* = −13→3
*T*_min_ = 0.675, *T*_max_ = 0.782	*k* = −5→13
1897 measured reflections	*l* = −12→12
847 independent reflections	

### Potassium sodium titanium iron tris(phosphate) (I) Refinement

**Table:**

Refinement on *F*^2^	'*w* = 1/[σ^2^(*F*_o_^2^) + (0.0292*P*)^2^ + 0.5767*P*] where *P* = (*F*_o_^2^ + 2*F*_c_^2^)/3'
Least-squares matrix: full	(Δ/σ)_max_ < 0.001
*R*[*F*^2^ > 2σ(*F*^2^)] = 0.025	Δρ_max_ = 0.48 e Å^−^^3^
*wR*(*F*^2^) = 0.064	Δρ_min_ = −0.37 e Å^−^^3^
*S* = 1.14	Extinction correction: SHELXL-2018/3 (Sheldrick 2015)
847 reflections	Extinction coefficient: 0.0042 (16)
63 parameters	Absolute structure: Flack *x* determined using 339 quotients [(*I*^+^)-(*I*^-^)]/[(*I*^+^)+(*I*^-^)] (Parsons *et al.*, 2013)
0 restraints	Absolute structure parameter: 0.02

### Potassium sodium titanium iron tris(phosphate) (I) Special details

**Table:**

Geometry. All esds (except the esd in the dihedral angle between two l.s. planes) are estimated using the full covariance matrix. The cell esds are taken into account individually in the estimation of esds in distances, angles and torsion angles; correlations between esds in cell parameters are only used when they are defined by crystal symmetry. An approximate (isotropic) treatment of cell esds is used for estimating esds involving l.s. planes.

### Potassium sodium titanium iron tris(phosphate) (I) Fractional atomic coordinates and isotropic or equivalent isotropic displacement parameters (Å^2^)

**Table:**

	*x*	*y*	*z*	*U*_iso_*/*U*_eq_	Occ. (<1)
Fe1	0.14303 (6)	0.14303 (6)	0.14303 (6)	0.0085 (3)	0.52 (3)
Ti1	0.14303 (6)	0.14303 (6)	0.14303 (6)	0.0085 (3)	0.48 (3)
Fe2	0.41389 (6)	0.41389 (6)	0.41389 (6)	0.0087 (3)	0.48 (3)
Ti2	0.41389 (6)	0.41389 (6)	0.41389 (6)	0.0087 (3)	0.52 (3)
K1	0.70712 (13)	0.70712 (13)	0.70712 (13)	0.0254 (7)	0.85 (2)
Na1	0.70712 (13)	0.70712 (13)	0.70712 (13)	0.0254 (7)	0.15 (2)
K2	0.93216 (12)	0.93216 (12)	0.93216 (12)	0.0228 (8)	0.80 (3)
Na2	0.93216 (12)	0.93216 (12)	0.93216 (12)	0.0228 (8)	0.20 (3)
P3	0.45810 (10)	0.22783 (10)	0.12639 (11)	0.0089 (3)	
O1	0.3106 (3)	0.2345 (3)	0.0792 (3)	0.0181 (7)	
O2	0.5477 (4)	0.2988 (4)	0.0217 (3)	0.0214 (8)	
O3	0.5021 (3)	0.0809 (3)	0.1494 (4)	0.0207 (7)	
O4	0.4787 (4)	0.3041 (4)	0.2590 (4)	0.0254 (9)	

### Potassium sodium titanium iron tris(phosphate) (I) Atomic displacement parameters (Å^2^)

**Table:**

	*U*^11^	*U*^22^	*U*^33^	*U*^12^	*U*^13^	*U*^23^
Fe1	0.0085 (3)	0.0085 (3)	0.0085 (3)	−0.0002 (2)	−0.0002 (2)	−0.0002 (2)
Ti1	0.0085 (3)	0.0085 (3)	0.0085 (3)	−0.0002 (2)	−0.0002 (2)	−0.0002 (2)
Fe2	0.0087 (3)	0.0087 (3)	0.0087 (3)	−0.0005 (2)	−0.0005 (2)	−0.0005 (2)
Ti2	0.0087 (3)	0.0087 (3)	0.0087 (3)	−0.0005 (2)	−0.0005 (2)	−0.0005 (2)
K1	0.0254 (7)	0.0254 (7)	0.0254 (7)	0.0004 (5)	0.0004 (5)	0.0004 (5)
Na1	0.0254 (7)	0.0254 (7)	0.0254 (7)	0.0004 (5)	0.0004 (5)	0.0004 (5)
K2	0.0228 (8)	0.0228 (8)	0.0228 (8)	−0.0021 (4)	−0.0021 (4)	−0.0021 (4)
Na2	0.0228 (8)	0.0228 (8)	0.0228 (8)	−0.0021 (4)	−0.0021 (4)	−0.0021 (4)
P3	0.0078 (5)	0.0098 (5)	0.0090 (5)	−0.0003 (3)	0.0014 (4)	0.0001 (4)
O1	0.0103 (14)	0.0218 (16)	0.0222 (17)	−0.0032 (12)	−0.0019 (12)	0.0080 (14)
O2	0.0190 (17)	0.0273 (17)	0.0178 (16)	0.0001 (14)	0.0060 (14)	0.0096 (14)
O3	0.0225 (16)	0.0123 (14)	0.0273 (17)	0.0070 (13)	0.0027 (14)	0.0027 (14)
O4	0.0278 (19)	0.029 (2)	0.0190 (17)	−0.0027 (15)	0.0019 (15)	−0.0148 (15)

### Potassium sodium titanium iron tris(phosphate) (I) Geometric parameters (Å, º)

**Table:**

Fe1—O2^i^	1.954 (3)	K1—O2^xviii^	3.019 (4)
Fe1—O2^ii^	1.954 (3)	K1—O4^xvi^	3.129 (4)
Fe1—O2^iii^	1.954 (3)	K1—O4^xvii^	3.129 (4)
Fe1—O1	1.976 (3)	K1—O4^xviii^	3.129 (4)
Fe1—O1^iv^	1.976 (3)	K1—P3^xvi^	3.4416 (16)
Fe1—O1^v^	1.976 (3)	K1—P3^xviii^	3.4416 (16)
Fe1—K2^vi^	3.587 (2)	K1—P3^xvii^	3.4416 (16)
Fe1—K1^vii^	3.7927 (9)	K2—O3^xvi^	2.854 (4)
Fe1—K1^viii^	3.7927 (9)	K2—O3^xvii^	2.854 (4)
Fe1—K1^ix^	3.7927 (9)	K2—O3^xviii^	2.854 (4)
Fe2—O3^x^	1.938 (3)	K2—O2^xix^	2.911 (4)
Fe2—O3^xi^	1.938 (3)	K2—O2^xx^	2.911 (4)
Fe2—O3^xii^	1.938 (3)	K2—O2^xxi^	2.911 (4)
Fe2—O4^v^	1.970 (3)	K2—O4^xvii^	3.007 (4)
Fe2—O4^iv^	1.970 (3)	K2—O4^xvi^	3.007 (4)
Fe2—O4	1.970 (3)	K2—O4^xviii^	3.007 (4)
Fe2—K2^xiii^	3.7237 (7)	K2—O4^xx^	3.231 (4)
Fe2—K2^xiv^	3.7237 (7)	K2—O4^xxi^	3.231 (4)
Fe2—K2^xv^	3.7237 (7)	K2—O4^xix^	3.231 (4)
K1—O1^xii^	2.830 (4)	P3—O4	1.516 (4)
K1—O1^x^	2.830 (4)	P3—O2	1.522 (3)
K1—O1^xi^	2.830 (4)	P3—O3	1.523 (3)
K1—O2^xvi^	3.019 (4)	P3—O1	1.523 (3)
K1—O2^xvii^	3.019 (4)		
			
O2^i^—Fe1—O2^ii^	89.19 (16)	O4^xvi^—K1—P3^xviii^	69.72 (7)
O2^i^—Fe1—O2^iii^	89.19 (16)	O4^xvii^—K1—P3^xviii^	103.33 (10)
O2^ii^—Fe1—O2^iii^	89.19 (16)	O4^xviii^—K1—P3^xviii^	26.12 (7)
O2^i^—Fe1—O1	177.99 (16)	P3^xvi^—K1—P3^xviii^	94.91 (5)
O2^ii^—Fe1—O1	88.89 (15)	O1^xii^—K1—P3^xvii^	94.57 (7)
O2^iii^—Fe1—O1	90.18 (14)	O1^x^—K1—P3^xvii^	79.17 (7)
O2^i^—Fe1—O1^iv^	88.88 (15)	O1^xi^—K1—P3^xvii^	169.22 (7)
O2^ii^—Fe1—O1^iv^	90.18 (14)	O2^xvi^—K1—P3^xvii^	108.29 (8)
O2^iii^—Fe1—O1^iv^	177.99 (16)	O2^xvii^—K1—P3^xvii^	26.23 (6)
O1—Fe1—O1^iv^	91.72 (14)	O2^xviii^—K1—P3^xvii^	115.08 (8)
O2^i^—Fe1—O1^v^	90.18 (14)	O4^xvi^—K1—P3^xvii^	103.33 (10)
O2^ii^—Fe1—O1^v^	177.99 (16)	O4^xvii^—K1—P3^xvii^	26.12 (7)
O2^iii^—Fe1—O1^v^	88.88 (15)	O4^xviii^—K1—P3^xvii^	69.72 (7)
O1—Fe1—O1^v^	91.71 (14)	P3^xvi^—K1—P3^xvii^	94.91 (5)
O1^iv^—Fe1—O1^v^	91.72 (14)	P3^xviii^—K1—P3^xvii^	94.91 (5)
O2^i^—Fe1—K2^vi^	54.17 (11)	O3^xvi^—K2—O3^xvii^	100.76 (10)
O2^ii^—Fe1—K2^vi^	54.17 (11)	O3^xvi^—K2—O3^xviii^	100.76 (10)
O2^iii^—Fe1—K2^vi^	54.17 (11)	O3^xvii^—K2—O3^xviii^	100.76 (10)
O1—Fe1—K2^vi^	124.04 (10)	O3^xvi^—K2—O2^xix^	100.42 (10)
O1^iv^—Fe1—K2^vi^	124.04 (10)	O3^xvii^—K2—O2^xix^	149.92 (11)
O1^v^—Fe1—K2^vi^	124.04 (10)	O3^xviii^—K2—O2^xix^	95.97 (10)
O2^i^—Fe1—K1^vii^	52.19 (11)	O3^xvi^—K2—O2^xx^	95.97 (10)
O2^ii^—Fe1—K1^vii^	131.75 (12)	O3^xvii^—K2—O2^xx^	100.42 (10)
O2^iii^—Fe1—K1^vii^	65.77 (11)	O3^xviii^—K2—O2^xx^	149.92 (11)
O1—Fe1—K1^vii^	129.13 (11)	O2^xix^—K2—O2^xx^	56.22 (11)
O1^iv^—Fe1—K1^vii^	113.38 (10)	O3^xvi^—K2—O2^xxi^	149.92 (11)
O1^v^—Fe1—K1^vii^	46.69 (10)	O3^xvii^—K2—O2^xxi^	95.97 (10)
K2^vi^—Fe1—K1^vii^	78.252 (17)	O3^xviii^—K2—O2^xxi^	100.42 (10)
O2^i^—Fe1—K1^viii^	65.77 (11)	O2^xix^—K2—O2^xxi^	56.22 (11)
O2^ii^—Fe1—K1^viii^	52.19 (11)	O2^xx^—K2—O2^xxi^	56.22 (11)
O2^iii^—Fe1—K1^viii^	131.75 (12)	O3^xvi^—K2—O4^xvii^	52.44 (10)
O1—Fe1—K1^viii^	113.38 (10)	O3^xvii^—K2—O4^xvii^	49.39 (9)
O1^iv^—Fe1—K1^viii^	46.69 (10)	O3^xviii^—K2—O4^xvii^	115.63 (12)
O1^v^—Fe1—K1^viii^	129.13 (11)	O2^xix^—K2—O4^xvii^	140.25 (11)
K2^vi^—Fe1—K1^viii^	78.252 (17)	O2^xx^—K2—O4^xvii^	94.39 (10)
K1^vii^—Fe1—K1^viii^	115.965 (12)	O2^xxi^—K2—O4^xvii^	132.45 (10)
O2^i^—Fe1—K1^ix^	131.75 (12)	O3^xvi^—K2—O4^xvi^	49.39 (9)
O2^ii^—Fe1—K1^ix^	65.77 (11)	O3^xvii^—K2—O4^xvi^	115.63 (12)
O2^iii^—Fe1—K1^ix^	52.19 (11)	O3^xviii^—K2—O4^xvi^	52.44 (10)
O1—Fe1—K1^ix^	46.69 (10)	O2^xix^—K2—O4^xvi^	94.39 (10)
O1^iv^—Fe1—K1^ix^	129.13 (11)	O2^xx^—K2—O4^xvi^	132.45 (10)
O1^v^—Fe1—K1^ix^	113.38 (10)	O2^xxi^—K2—O4^xvi^	140.25 (11)
K2^vi^—Fe1—K1^ix^	78.252 (17)	O4^xvii^—K2—O4^xvi^	87.30 (11)
K1^vii^—Fe1—K1^ix^	115.965 (12)	O3^xvi^—K2—O4^xviii^	115.63 (12)
K1^viii^—Fe1—K1^ix^	115.965 (12)	O3^xvii^—K2—O4^xviii^	52.44 (10)
O3^x^—Fe2—O3^xi^	92.72 (15)	O3^xviii^—K2—O4^xviii^	49.39 (9)
O3^x^—Fe2—O3^xii^	92.72 (15)	O2^xix^—K2—O4^xviii^	132.45 (10)
O3^xi^—Fe2—O3^xii^	92.72 (15)	O2^xx^—K2—O4^xviii^	140.25 (11)
O3^x^—Fe2—O4^v^	171.85 (17)	O2^xxi^—K2—O4^xviii^	94.39 (10)
O3^xi^—Fe2—O4^v^	83.11 (16)	O4^xvii^—K2—O4^xviii^	87.30 (11)
O3^xii^—Fe2—O4^v^	94.47 (15)	O4^xvi^—K2—O4^xviii^	87.30 (11)
O3^x^—Fe2—O4^iv^	94.47 (15)	O3^xvi^—K2—O4^xx^	55.86 (9)
O3^xi^—Fe2—O4^iv^	171.85 (17)	O3^xvii^—K2—O4^xx^	85.99 (9)
O3^xii^—Fe2—O4^iv^	83.11 (16)	O3^xviii^—K2—O4^xx^	156.61 (10)
O4^v^—Fe2—O4^iv^	90.22 (16)	O2^xix^—K2—O4^xx^	88.46 (10)
O3^x^—Fe2—O4	83.11 (16)	O2^xx^—K2—O4^xx^	46.20 (9)
O3^xi^—Fe2—O4	94.47 (15)	O2^xxi^—K2—O4^xx^	101.11 (10)
O3^xii^—Fe2—O4	171.85 (17)	O4^xvii^—K2—O4^xx^	53.02 (13)
O4^v^—Fe2—O4	90.22 (16)	O4^xvi^—K2—O4^xx^	104.40 (2)
O4^iv^—Fe2—O4	90.22 (16)	O4^xviii^—K2—O4^xx^	137.03 (8)
O3^x^—Fe2—K2^xiii^	127.93 (11)	O3^xvi^—K2—O4^xxi^	156.61 (10)
O3^xi^—Fe2—K2^xiii^	118.56 (11)	O3^xvii^—K2—O4^xxi^	55.86 (9)
O3^xii^—Fe2—K2^xiii^	48.96 (11)	O3^xviii^—K2—O4^xxi^	85.99 (9)
O4^v^—Fe2—K2^xiii^	60.13 (13)	O2^xix^—K2—O4^xxi^	101.11 (10)
O4^iv^—Fe2—K2^xiii^	53.60 (12)	O2^xx^—K2—O4^xxi^	88.46 (10)
O4—Fe2—K2^xiii^	129.40 (12)	O2^xxi^—K2—O4^xxi^	46.20 (9)
O3^x^—Fe2—K2^xiv^	118.56 (11)	O4^xvii^—K2—O4^xxi^	104.40 (2)
O3^xi^—Fe2—K2^xiv^	48.96 (11)	O4^xvi^—K2—O4^xxi^	137.03 (8)
O3^xii^—Fe2—K2^xiv^	127.93 (11)	O4^xviii^—K2—O4^xxi^	53.02 (13)
O4^v^—Fe2—K2^xiv^	53.60 (12)	O4^xx^—K2—O4^xxi^	115.75 (5)
O4^iv^—Fe2—K2^xiv^	129.40 (12)	O3^xvi^—K2—O4^xix^	85.99 (9)
O4—Fe2—K2^xiv^	60.13 (12)	O3^xvii^—K2—O4^xix^	156.61 (10)
K2^xiii^—Fe2—K2^xiv^	113.261 (15)	O3^xviii^—K2—O4^xix^	55.86 (9)
O3^x^—Fe2—K2^xv^	48.96 (11)	O2^xix^—K2—O4^xix^	46.20 (9)
O3^xi^—Fe2—K2^xv^	127.93 (11)	O2^xx^—K2—O4^xix^	101.11 (10)
O3^xii^—Fe2—K2^xv^	118.56 (11)	O2^xxi^—K2—O4^xix^	88.46 (10)
O4^v^—Fe2—K2^xv^	129.40 (12)	O4^xvii^—K2—O4^xix^	137.03 (8)
O4^iv^—Fe2—K2^xv^	60.13 (12)	O4^xvi^—K2—O4^xix^	53.02 (13)
O4—Fe2—K2^xv^	53.60 (12)	O4^xviii^—K2—O4^xix^	104.40 (2)
K2^xiii^—Fe2—K2^xv^	113.261 (15)	O4^xx^—K2—O4^xix^	115.75 (5)
K2^xiv^—Fe2—K2^xv^	113.261 (15)	O4^xxi^—K2—O4^xix^	115.75 (5)
O1^xii^—K1—O1^x^	92.24 (11)	O4—P3—O2	106.1 (2)
O1^xii^—K1—O1^xi^	92.24 (12)	O4—P3—O3	107.6 (2)
O1^x^—K1—O1^xi^	92.24 (11)	O2—P3—O3	111.7 (2)
O1^xii^—K1—O2^xvi^	56.73 (9)	O4—P3—O1	111.6 (2)
O1^x^—K1—O2^xvi^	148.02 (12)	O2—P3—O1	109.0 (2)
O1^xi^—K1—O2^xvi^	82.44 (10)	O3—P3—O1	110.81 (19)
O1^xii^—K1—O2^xvii^	82.44 (10)	O4—P3—K2^xiv^	70.72 (16)
O1^x^—K1—O2^xvii^	56.73 (9)	O2—P3—K2^xiv^	58.63 (14)
O1^xi^—K1—O2^xvii^	148.02 (12)	O3—P3—K2^xiv^	167.81 (14)
O2^xvi^—K1—O2^xvii^	118.99 (3)	O1—P3—K2^xiv^	80.51 (13)
O1^xii^—K1—O2^xviii^	148.02 (12)	O4—P3—K1^xv^	65.34 (15)
O1^x^—K1—O2^xviii^	82.44 (10)	O2—P3—K1^xv^	61.21 (14)
O1^xi^—K1—O2^xviii^	56.73 (9)	O3—P3—K1^xv^	82.59 (14)
O2^xvi^—K1—O2^xviii^	118.99 (3)	O1—P3—K1^xv^	166.21 (14)
O2^xvii^—K1—O2^xviii^	118.99 (3)	K2^xiv^—P3—K1^xv^	85.88 (3)
O1^xii^—K1—O4^xvi^	103.04 (9)	O4—P3—K2^xv^	56.80 (16)
O1^x^—K1—O4^xvi^	164.18 (10)	O2—P3—K2^xv^	126.68 (15)
O1^xi^—K1—O4^xvi^	83.19 (10)	O3—P3—K2^xv^	50.98 (15)
O2^xvi^—K1—O4^xvi^	46.48 (9)	O1—P3—K2^xv^	124.35 (14)
O2^xvii^—K1—O4^xvi^	128.76 (12)	K2^xiv^—P3—K2^xv^	126.85 (4)
O2^xviii^—K1—O4^xvi^	82.41 (10)	K1^xv^—P3—K2^xv^	66.30 (5)
O1^xii^—K1—O4^xvii^	83.19 (10)	O4—P3—K1^ix^	148.79 (17)
O1^x^—K1—O4^xvii^	103.04 (9)	O2—P3—K1^ix^	71.17 (14)
O1^xi^—K1—O4^xvii^	164.18 (10)	O3—P3—K1^ix^	101.86 (15)
O2^xvi^—K1—O4^xvii^	82.41 (10)	O1—P3—K1^ix^	46.23 (13)
O2^xvii^—K1—O4^xvii^	46.48 (9)	K2^xiv^—P3—K1^ix^	82.53 (3)
O2^xviii^—K1—O4^xvii^	128.76 (12)	K1^xv^—P3—K1^ix^	129.78 (5)
O4^xvi^—K1—O4^xvii^	83.10 (12)	K2^xv^—P3—K1^ix^	149.91 (4)
O1^xii^—K1—O4^xviii^	164.18 (10)	P3—O1—Fe1	132.5 (2)
O1^x^—K1—O4^xviii^	83.19 (10)	P3—O1—K1^ix^	110.90 (17)
O1^xi^—K1—O4^xviii^	103.04 (9)	Fe1—O1—K1^ix^	102.77 (13)
O2^xvi^—K1—O4^xviii^	128.76 (12)	P3—O2—Fe1^xxii^	165.9 (2)
O2^xvii^—K1—O4^xviii^	82.41 (10)	P3—O2—K2^xiv^	94.85 (16)
O2^xviii^—K1—O4^xviii^	46.48 (9)	Fe1^xxii^—O2—K2^xiv^	92.87 (13)
O4^xvi^—K1—O4^xviii^	83.10 (12)	P3—O2—K1^xv^	92.56 (16)
O4^xvii^—K1—O4^xviii^	83.10 (12)	Fe1^xxii^—O2—K1^xv^	97.07 (13)
O1^xii^—K1—P3^xvi^	79.17 (7)	K2^xiv^—O2—K1^xv^	103.55 (11)
O1^x^—K1—P3^xvi^	169.22 (7)	P3—O3—Fe2^ix^	151.0 (2)
O1^xi^—K1—P3^xvi^	94.57 (7)	P3—O3—K2^xv^	104.53 (18)
O2^xvi^—K1—P3^xvi^	26.23 (6)	Fe2^ix^—O3—K2^xv^	100.23 (13)
O2^xvii^—K1—P3^xvi^	115.08 (8)	P3—O4—Fe2	152.9 (3)
O2^xviii^—K1—P3^xvi^	108.29 (8)	P3—O4—K2^xv^	98.25 (18)
O4^xvi^—K1—P3^xvi^	26.12 (7)	Fe2—O4—K2^xv^	94.57 (14)
O4^xvii^—K1—P3^xvi^	69.72 (7)	P3—O4—K1^xv^	88.54 (16)
O4^xviii^—K1—P3^xvi^	103.33 (10)	Fe2—O4—K1^xv^	117.62 (15)
O1^xii^—K1—P3^xviii^	169.22 (7)	K2^xv^—O4—K1^xv^	77.17 (10)
O1^x^—K1—P3^xviii^	94.57 (7)	P3—O4—K2^xiv^	83.00 (16)
O1^xi^—K1—P3^xviii^	79.17 (7)	Fe2—O4—K2^xiv^	87.94 (14)
O2^xvi^—K1—P3^xviii^	115.08 (8)	K2^xv^—O4—K2^xiv^	171.21 (14)
O2^xvii^—K1—P3^xviii^	108.29 (8)	K1^xv^—O4—K2^xiv^	94.20 (11)
O2^xviii^—K1—P3^xviii^	26.23 (6)		

Symmetry codes: (i) −*z*, *x*−1/2, −*y*+1/2; (ii) −*y*+1/2, −*z*, *x*−1/2; (iii) *x*−1/2, −*y*+1/2, −*z*; (iv) *y*, *z*, *x*; (v) *z*, *x*, *y*; (vi) *x*−1, *y*−1, *z*−1; (vii) −*x*+1/2, −*y*+1, *z*−1/2; (viii) *x*−1/2, −*y*+1/2, −*z*+1; (ix) −*x*+1, *y*−1/2, −*z*+1/2; (x) *y*+1/2, −*z*+1/2, −*x*+1; (xi) −*x*+1, *y*+1/2, −*z*+1/2; (xii) −*z*+1/2, −*x*+1, *y*+1/2; (xiii) −*x*+1, *y*−1/2, −*z*+3/2; (xiv) *x*−1/2, −*y*+3/2, −*z*+1; (xv) −*x*+3/2, −*y*+1, *z*−1/2; (xvi) *z*+1/2, −*x*+3/2, −*y*+1; (xvii) −*y*+1, *z*+1/2, −*x*+3/2; (xviii) −*x*+3/2, −*y*+1, *z*+1/2; (xix) *x*+1/2, −*y*+3/2, −*z*+1; (xx) −*z*+1, *x*+1/2, −*y*+3/2; (xxi) −*y*+3/2, −*z*+1, *x*+1/2; (xxii) *x*+1/2, −*y*+1/2, −*z*.

### Potassium sodium titanium iron tris(phosphate) (II) Crystal data

**Table:**

K_0.97_Na_1.03_Ti_1.26_Fe_0.74_(PO_4_)_3_	*D*_x_ = 3.168 Mg m^−^^3^
*M**_r_* = 448.16	Mo *K*α radiation, λ = 0.71073 Å
Cubic, *P*2_1_3	Cell parameters from 10546 reflections
Hall symbol: P 2ac 2ab 3	θ = 2.9–29.0°
*a* = 9.7945 (1) Å	µ = 3.27 mm^−^^1^
*V* = 939.61 (3) Å^3^	*T* = 293 K
*Z* = 4	Tetrahedron, violet
*F*(000) = 870.9	0.15 × 0.11 × 0.08 mm

### Potassium sodium titanium iron tris(phosphate) (II) Data collection

**Table:**

Oxford Diffraction Xcalibur-3 diffractometer	833 reflections with *I* > 2σ(*I*)
Graphite monochromator	*R*_int_ = 0.026
φ and ω scans	θ_max_ = 29.0°, θ_min_ = 2.9°
Absorption correction: multi-scan (Blessing, 1995)	*h* = −12→13
*T*_min_ = 0.622, *T*_max_ = 0.835	*k* = −13→13
10546 measured reflections	*l* = −13→13
837 independent reflections	

### Potassium sodium titanium iron tris(phosphate) (II) Refinement

**Table:**

Refinement on *F*^2^	'*w* = 1/[σ^2^(*F*_o_^2^) + (0.0186*P*)^2^ + 1.1348*P*] where *P* = (*F*_o_^2^ + 2*F*_c_^2^)/3'
Least-squares matrix: full	(Δ/σ)_max_ < 0.001
*R*[*F*^2^ > 2σ(*F*^2^)] = 0.016	Δρ_max_ = 0.28 e Å^−^^3^
*wR*(*F*^2^) = 0.043	Δρ_min_ = −0.27 e Å^−^^3^
*S* = 1.12	Extinction correction: SHELXL-2018/3 (Sheldrick 2015)
837 reflections	Extinction coefficient: 0.0015 (10)
63 parameters	Absolute structure: Flack *x* determined using 349 quotients [(*I*^+^)-(*I*^-^)]/[(*I*^+^)+(*I*^-^)] (Parsons *et al.*, 2013)
0 restraints	Absolute structure parameter: 0.02

### Potassium sodium titanium iron tris(phosphate) (II) Special details

**Table:**

Geometry. All esds (except the esd in the dihedral angle between two l.s. planes) are estimated using the full covariance matrix. The cell esds are taken into account individually in the estimation of esds in distances, angles and torsion angles; correlations between esds in cell parameters are only used when they are defined by crystal symmetry. An approximate (isotropic) treatment of cell esds is used for estimating esds involving l.s. planes.

### Potassium sodium titanium iron tris(phosphate) (II) Fractional atomic coordinates and isotropic or equivalent isotropic displacement parameters (Å^2^)

**Table:**

	*x*	*y*	*z*	*U*_iso_*/*U*_eq_	Occ. (<1)
Fe1	0.14198 (4)	0.14198 (4)	0.14198 (4)	0.0079 (2)	0.39 (2)
Ti1	0.14198 (4)	0.14198 (4)	0.14198 (4)	0.0079 (2)	0.61 (2)
Fe2	0.41334 (4)	0.41334 (4)	0.41334 (4)	0.0079 (2)	0.35 (2)
Ti2	0.41334 (4)	0.41334 (4)	0.41334 (4)	0.0079 (2)	0.65 (2)
K1	0.70732 (10)	0.70732 (10)	0.70732 (10)	0.0266 (6)	0.676 (18)
Na1	0.70732 (10)	0.70732 (10)	0.70732 (10)	0.0266 (6)	0.324 (18)
K2	0.93159 (11)	0.93159 (11)	0.93159 (11)	0.0254 (8)	0.294 (19)
Na2	0.93159 (11)	0.93159 (11)	0.93159 (11)	0.0254 (8)	0.706 (19)
P3	0.45787 (7)	0.22778 (7)	0.12657 (7)	0.00815 (19)	
O1	0.3100 (2)	0.2337 (3)	0.0789 (2)	0.0210 (5)	
O2	0.5478 (3)	0.2989 (3)	0.0220 (3)	0.0266 (6)	
O3	0.5024 (3)	0.0810 (2)	0.1492 (3)	0.0269 (5)	
O4	0.4786 (3)	0.3034 (3)	0.2602 (3)	0.0313 (6)	

### Potassium sodium titanium iron tris(phosphate) (II) Atomic displacement parameters (Å^2^)

**Table:**

	*U*^11^	*U*^22^	*U*^33^	*U*^12^	*U*^13^	*U*^23^
Fe1	0.0079 (2)	0.0079 (2)	0.0079 (2)	0.00028 (15)	0.00028 (15)	0.00028 (15)
Ti1	0.0079 (2)	0.0079 (2)	0.0079 (2)	0.00028 (15)	0.00028 (15)	0.00028 (15)
Fe2	0.0079 (2)	0.0079 (2)	0.0079 (2)	−0.00052 (15)	−0.00052 (15)	−0.00052 (15)
Ti2	0.0079 (2)	0.0079 (2)	0.0079 (2)	−0.00052 (15)	−0.00052 (15)	−0.00052 (15)
K1	0.0266 (6)	0.0266 (6)	0.0266 (6)	0.0015 (4)	0.0015 (4)	0.0015 (4)
Na1	0.0266 (6)	0.0266 (6)	0.0266 (6)	0.0015 (4)	0.0015 (4)	0.0015 (4)
K2	0.0254 (8)	0.0254 (8)	0.0254 (8)	−0.0021 (4)	−0.0021 (4)	−0.0021 (4)
Na2	0.0254 (8)	0.0254 (8)	0.0254 (8)	−0.0021 (4)	−0.0021 (4)	−0.0021 (4)
P3	0.0075 (3)	0.0087 (3)	0.0083 (3)	−0.0003 (2)	0.0015 (2)	−0.0007 (2)
O1	0.0088 (9)	0.0299 (12)	0.0242 (12)	−0.0033 (8)	−0.0020 (8)	0.0089 (10)
O2	0.0197 (11)	0.0361 (14)	0.0241 (12)	−0.0014 (10)	0.0088 (10)	0.0136 (11)
O3	0.0263 (12)	0.0128 (10)	0.0415 (14)	0.0088 (9)	0.0053 (11)	0.0032 (11)
O4	0.0333 (14)	0.0373 (15)	0.0232 (12)	−0.0027 (12)	0.0014 (11)	−0.0206 (11)

### Potassium sodium titanium iron tris(phosphate) (II) Geometric parameters (Å, º)

**Table:**

Fe1—O2^i^	1.940 (2)	K1—O2^xviii^	3.009 (3)
Fe1—O2^ii^	1.940 (2)	K1—O4^xvii^	3.122 (3)
Fe1—O2^iii^	1.940 (2)	K1—O4^xviii^	3.122 (3)
Fe1—O1	1.974 (2)	K1—O4^xvi^	3.122 (3)
Fe1—O1^iv^	1.974 (2)	K1—P3^xviii^	3.4327 (11)
Fe1—O1^v^	1.974 (2)	K1—P3^xvii^	3.4327 (11)
Fe1—K2^vi^	3.569 (2)	K1—P3^xvi^	3.4327 (11)
Fe1—K1^vii^	3.7806 (7)	K2—O3^xvii^	2.843 (3)
Fe1—K1^viii^	3.7806 (7)	K2—O3^xvi^	2.843 (3)
Fe1—K1^ix^	3.7806 (7)	K2—O3^xviii^	2.843 (3)
Fe2—O3^x^	1.938 (2)	K2—O2^xix^	2.910 (3)
Fe2—O3^xi^	1.938 (2)	K2—O2^xx^	2.910 (3)
Fe2—O3^xii^	1.938 (2)	K2—O2^xxi^	2.910 (3)
Fe2—O4	1.954 (2)	K2—O4^xvii^	2.982 (4)
Fe2—O4^iv^	1.954 (2)	K2—O4^xvi^	2.982 (4)
Fe2—O4^v^	1.954 (2)	K2—O4^xviii^	2.982 (4)
Fe2—K2^xiii^	3.7084 (6)	K2—O4^xx^	3.237 (3)
Fe2—K2^xiv^	3.7084 (6)	K2—O4^xix^	3.237 (3)
Fe2—K2^xv^	3.7084 (6)	K2—O4^xxi^	3.237 (3)
K1—O1^xi^	2.820 (3)	P3—O4	1.517 (3)
K1—O1^xii^	2.820 (3)	P3—O3	1.518 (2)
K1—O1^x^	2.820 (3)	P3—O2	1.520 (2)
K1—O2^xvi^	3.009 (3)	P3—O1	1.523 (2)
K1—O2^xvii^	3.009 (3)		
			
O2^i^—Fe1—O2^ii^	89.72 (12)	O4^xvii^—K1—P3^xvii^	26.22 (5)
O2^i^—Fe1—O2^iii^	89.72 (12)	O4^xviii^—K1—P3^xvii^	103.21 (8)
O2^ii^—Fe1—O2^iii^	89.72 (12)	O4^xvi^—K1—P3^xvii^	69.59 (5)
O2^i^—Fe1—O1	178.52 (12)	P3^xviii^—K1—P3^xvii^	94.92 (4)
O2^ii^—Fe1—O1	88.81 (11)	O1^xi^—K1—P3^xvi^	94.74 (5)
O2^iii^—Fe1—O1	90.09 (10)	O1^xii^—K1—P3^xvi^	79.13 (5)
O2^i^—Fe1—O1^iv^	90.09 (10)	O1^x^—K1—P3^xvi^	169.06 (5)
O2^ii^—Fe1—O1^iv^	178.52 (12)	O2^xvi^—K1—P3^xvi^	26.25 (5)
O2^iii^—Fe1—O1^iv^	88.81 (11)	O2^xvii^—K1—P3^xvi^	108.35 (6)
O1—Fe1—O1^iv^	91.38 (10)	O2^xviii^—K1—P3^xvi^	115.09 (6)
O2^i^—Fe1—O1^v^	88.81 (11)	O4^xvii^—K1—P3^xvi^	103.21 (8)
O2^ii^—Fe1—O1^v^	90.09 (10)	O4^xviii^—K1—P3^xvi^	69.59 (5)
O2^iii^—Fe1—O1^v^	178.52 (12)	O4^xvi^—K1—P3^xvi^	26.22 (5)
O1—Fe1—O1^v^	91.38 (10)	P3^xviii^—K1—P3^xvi^	94.92 (4)
O1^iv^—Fe1—O1^v^	91.38 (10)	P3^xvii^—K1—P3^xvi^	94.92 (4)
O2^i^—Fe1—K2^vi^	54.54 (9)	O3^xvii^—K2—O3^xvi^	100.89 (8)
O2^ii^—Fe1—K2^vi^	54.54 (9)	O3^xvii^—K2—O3^xviii^	100.89 (8)
O2^iii^—Fe1—K2^vi^	54.54 (9)	O3^xvi^—K2—O3^xviii^	100.89 (8)
O1—Fe1—K2^vi^	124.28 (7)	O3^xvii^—K2—O2^xix^	149.75 (9)
O1^iv^—Fe1—K2^vi^	124.28 (7)	O3^xvi^—K2—O2^xix^	95.89 (7)
O1^v^—Fe1—K2^vi^	124.28 (7)	O3^xviii^—K2—O2^xix^	100.42 (8)
O2^i^—Fe1—K1^vii^	132.34 (9)	O3^xvii^—K2—O2^xx^	95.89 (7)
O2^ii^—Fe1—K1^vii^	66.00 (8)	O3^xvi^—K2—O2^xx^	100.42 (8)
O2^iii^—Fe1—K1^vii^	52.14 (8)	O3^xviii^—K2—O2^xx^	149.75 (9)
O1—Fe1—K1^vii^	46.70 (7)	O2^xix^—K2—O2^xx^	56.11 (8)
O1^iv^—Fe1—K1^vii^	113.14 (8)	O3^xvii^—K2—O2^xxi^	100.42 (8)
O1^v^—Fe1—K1^vii^	129.02 (8)	O3^xvi^—K2—O2^xxi^	149.75 (9)
K2^vi^—Fe1—K1^vii^	78.502 (13)	O3^xviii^—K2—O2^xxi^	95.89 (7)
O2^i^—Fe1—K1^viii^	66.00 (8)	O2^xix^—K2—O2^xxi^	56.11 (8)
O2^ii^—Fe1—K1^viii^	52.14 (8)	O2^xx^—K2—O2^xxi^	56.11 (8)
O2^iii^—Fe1—K1^viii^	132.34 (9)	O3^xvii^—K2—O4^xvii^	49.57 (7)
O1—Fe1—K1^viii^	113.14 (8)	O3^xvi^—K2—O4^xvii^	115.99 (10)
O1^iv^—Fe1—K1^viii^	129.02 (8)	O3^xviii^—K2—O4^xvii^	52.41 (7)
O1^v^—Fe1—K1^viii^	46.70 (7)	O2^xix^—K2—O4^xvii^	140.03 (8)
K2^vi^—Fe1—K1^viii^	78.502 (13)	O2^xx^—K2—O4^xvii^	132.27 (7)
K1^vii^—Fe1—K1^viii^	116.129 (8)	O2^xxi^—K2—O4^xvii^	94.19 (7)
O2^i^—Fe1—K1^ix^	52.14 (8)	O3^xvii^—K2—O4^xvi^	52.41 (7)
O2^ii^—Fe1—K1^ix^	132.34 (9)	O3^xvi^—K2—O4^xvi^	49.57 (7)
O2^iii^—Fe1—K1^ix^	66.00 (8)	O3^xviii^—K2—O4^xvi^	115.99 (10)
O1—Fe1—K1^ix^	129.02 (8)	O2^xix^—K2—O4^xvi^	132.27 (7)
O1^iv^—Fe1—K1^ix^	46.70 (7)	O2^xx^—K2—O4^xvi^	94.19 (7)
O1^v^—Fe1—K1^ix^	113.14 (8)	O2^xxi^—K2—O4^xvi^	140.03 (8)
K2^vi^—Fe1—K1^ix^	78.502 (13)	O4^xvii^—K2—O4^xvi^	87.67 (9)
K1^vii^—Fe1—K1^ix^	116.129 (8)	O3^xvii^—K2—O4^xviii^	115.99 (10)
K1^viii^—Fe1—K1^ix^	116.129 (8)	O3^xvi^—K2—O4^xviii^	52.41 (7)
O3^x^—Fe2—O3^xi^	92.37 (12)	O3^xviii^—K2—O4^xviii^	49.57 (7)
O3^x^—Fe2—O3^xii^	92.37 (12)	O2^xix^—K2—O4^xviii^	94.19 (7)
O3^xi^—Fe2—O3^xii^	92.37 (12)	O2^xx^—K2—O4^xviii^	140.03 (8)
O3^x^—Fe2—O4	94.89 (12)	O2^xxi^—K2—O4^xviii^	132.27 (8)
O3^xi^—Fe2—O4	171.47 (13)	O4^xvii^—K2—O4^xviii^	87.67 (9)
O3^xii^—Fe2—O4	82.87 (13)	O4^xvi^—K2—O4^xviii^	87.67 (9)
O3^x^—Fe2—O4^iv^	82.87 (13)	O3^xvii^—K2—O4^xx^	55.81 (6)
O3^xi^—Fe2—O4^iv^	94.90 (12)	O3^xvi^—K2—O4^xx^	86.02 (7)
O3^xii^—Fe2—O4^iv^	171.46 (13)	O3^xviii^—K2—O4^xx^	156.68 (8)
O4—Fe2—O4^iv^	90.46 (12)	O2^xix^—K2—O4^xx^	100.98 (8)
O3^x^—Fe2—O4^v^	171.46 (13)	O2^xx^—K2—O4^xx^	46.19 (7)
O3^xi^—Fe2—O4^v^	82.87 (13)	O2^xxi^—K2—O4^xx^	88.43 (8)
O3^xii^—Fe2—O4^v^	94.90 (12)	O4^xvii^—K2—O4^xx^	104.497 (19)
O4—Fe2—O4^v^	90.46 (12)	O4^xvi^—K2—O4^xx^	52.80 (10)
O4^iv^—Fe2—O4^v^	90.46 (12)	O4^xviii^—K2—O4^xx^	137.10 (6)
O3^x^—Fe2—K2^xiii^	118.47 (8)	O3^xvii^—K2—O4^xix^	156.68 (8)
O3^xi^—Fe2—K2^xiii^	49.04 (9)	O3^xvi^—K2—O4^xix^	55.81 (6)
O3^xii^—Fe2—K2^xiii^	127.81 (8)	O3^xviii^—K2—O4^xix^	86.02 (7)
O4—Fe2—K2^xiii^	129.70 (9)	O2^xix^—K2—O4^xix^	46.19 (7)
O4^iv^—Fe2—K2^xiii^	60.69 (10)	O2^xx^—K2—O4^xix^	88.43 (8)
O4^v^—Fe2—K2^xiii^	53.21 (10)	O2^xxi^—K2—O4^xix^	100.98 (8)
O3^x^—Fe2—K2^xiv^	127.81 (8)	O4^xvii^—K2—O4^xix^	137.10 (6)
O3^xi^—Fe2—K2^xiv^	118.47 (8)	O4^xvi^—K2—O4^xix^	104.497 (19)
O3^xii^—Fe2—K2^xiv^	49.04 (9)	O4^xviii^—K2—O4^xix^	52.80 (10)
O4—Fe2—K2^xiv^	53.21 (10)	O4^xx^—K2—O4^xix^	115.71 (4)
O4^iv^—Fe2—K2^xiv^	129.70 (9)	O3^xvii^—K2—O4^xxi^	86.02 (7)
O4^v^—Fe2—K2^xiv^	60.69 (10)	O3^xvi^—K2—O4^xxi^	156.68 (8)
K2^xiii^—Fe2—K2^xiv^	113.409 (11)	O3^xviii^—K2—O4^xxi^	55.81 (6)
O3^x^—Fe2—K2^xv^	49.04 (9)	O2^xix^—K2—O4^xxi^	88.43 (8)
O3^xi^—Fe2—K2^xv^	127.81 (8)	O2^xx^—K2—O4^xxi^	100.98 (8)
O3^xii^—Fe2—K2^xv^	118.47 (8)	O2^xxi^—K2—O4^xxi^	46.19 (7)
O4—Fe2—K2^xv^	60.69 (10)	O4^xvii^—K2—O4^xxi^	52.80 (10)
O4^iv^—Fe2—K2^xv^	53.21 (10)	O4^xvi^—K2—O4^xxi^	137.10 (6)
O4^v^—Fe2—K2^xv^	129.70 (9)	O4^xviii^—K2—O4^xxi^	104.497 (19)
K2^xiii^—Fe2—K2^xv^	113.409 (11)	O4^xx^—K2—O4^xxi^	115.71 (4)
K2^xiv^—Fe2—K2^xv^	113.409 (11)	O4^xix^—K2—O4^xxi^	115.71 (4)
O1^xi^—K1—O1^xii^	92.11 (9)	O4—P3—O3	107.34 (18)
O1^xi^—K1—O1^x^	92.11 (9)	O4—P3—O2	106.27 (16)
O1^xii^—K1—O1^x^	92.11 (9)	O3—P3—O2	111.46 (16)
O1^xi^—K1—O2^xvi^	82.55 (7)	O4—P3—O1	111.89 (15)
O1^xii^—K1—O2^xvi^	56.64 (6)	O3—P3—O1	110.74 (14)
O1^x^—K1—O2^xvi^	147.84 (9)	O2—P3—O1	109.06 (14)
O1^xi^—K1—O2^xvii^	56.64 (6)	O4—P3—K2^xv^	71.04 (13)
O1^xii^—K1—O2^xvii^	147.84 (9)	O3—P3—K2^xv^	167.62 (11)
O1^x^—K1—O2^xvii^	82.55 (7)	O2—P3—K2^xv^	58.68 (11)
O2^xvi^—K1—O2^xvii^	119.008 (19)	O1—P3—K2^xv^	80.72 (10)
O1^xi^—K1—O2^xviii^	147.84 (9)	O4—P3—K1^xiv^	65.37 (11)
O1^xii^—K1—O2^xviii^	82.55 (7)	O3—P3—K1^xiv^	82.38 (11)
O1^x^—K1—O2^xviii^	56.64 (6)	O2—P3—K1^xiv^	61.12 (11)
O2^xvi^—K1—O2^xviii^	119.008 (19)	O1—P3—K1^xiv^	166.43 (11)
O2^xvii^—K1—O2^xviii^	119.008 (19)	K2^xv^—P3—K1^xiv^	85.93 (3)
O1^xi^—K1—O4^xvii^	103.13 (7)	O4—P3—K2^xiv^	56.40 (13)
O1^xii^—K1—O4^xvii^	164.24 (7)	O3—P3—K2^xiv^	51.08 (12)
O1^x^—K1—O4^xvii^	83.46 (8)	O2—P3—K2^xiv^	126.42 (11)
O2^xvi^—K1—O4^xvii^	128.67 (9)	O1—P3—K2^xiv^	124.51 (10)
O2^xvii^—K1—O4^xvii^	46.65 (7)	K2^xv^—P3—K2^xiv^	126.74 (3)
O2^xviii^—K1—O4^xvii^	82.39 (7)	K1^xiv^—P3—K2^xiv^	66.11 (4)
O1^xi^—K1—O4^xviii^	164.24 (7)	O4—P3—K1^vii^	149.07 (13)
O1^xii^—K1—O4^xviii^	83.46 (8)	O3—P3—K1^vii^	101.87 (12)
O1^x^—K1—O4^xviii^	103.13 (7)	O2—P3—K1^vii^	71.24 (11)
O2^xvi^—K1—O4^xviii^	82.39 (7)	O1—P3—K1^vii^	46.09 (9)
O2^xvii^—K1—O4^xviii^	128.67 (9)	K2^xv^—P3—K1^vii^	82.54 (3)
O2^xviii^—K1—O4^xviii^	46.65 (7)	K1^xiv^—P3—K1^vii^	129.74 (3)
O4^xvii^—K1—O4^xviii^	82.84 (10)	K2^xiv^—P3—K1^vii^	150.05 (3)
O1^xi^—K1—O4^xvi^	83.46 (8)	P3—O1—Fe1	132.78 (15)
O1^xii^—K1—O4^xvi^	103.13 (7)	P3—O1—K1^vii^	111.02 (12)
O1^x^—K1—O4^xvi^	164.24 (7)	Fe1—O1—K1^vii^	102.66 (9)
O2^xvi^—K1—O4^xvi^	46.65 (7)	P3—O2—Fe1^xxii^	165.93 (19)
O2^xvii^—K1—O4^xvi^	82.39 (7)	P3—O2—K2^xv^	94.82 (12)
O2^xviii^—K1—O4^xvi^	128.67 (9)	Fe1^xxii^—O2—K2^xv^	92.57 (10)
O4^xvii^—K1—O4^xvi^	82.84 (10)	P3—O2—K1^xiv^	92.63 (12)
O4^xviii^—K1—O4^xvi^	82.84 (10)	Fe1^xxii^—O2—K1^xiv^	97.25 (10)
O1^xi^—K1—P3^xviii^	169.06 (5)	K2^xv^—O2—K1^xiv^	103.64 (9)
O1^xii^—K1—P3^xviii^	94.74 (5)	P3—O3—Fe2^vii^	151.51 (19)
O1^x^—K1—P3^xviii^	79.13 (5)	P3—O3—K2^xiv^	104.38 (14)
O2^xvi^—K1—P3^xviii^	108.35 (6)	Fe2^vii^—O3—K2^xiv^	100.00 (10)
O2^xvii^—K1—P3^xviii^	115.09 (6)	P3—O4—Fe2	152.6 (2)
O2^xviii^—K1—P3^xviii^	26.25 (5)	P3—O4—K2^xiv^	98.53 (14)
O4^xvii^—K1—P3^xviii^	69.59 (5)	Fe2—O4—K2^xiv^	95.14 (11)
O4^xviii^—K1—P3^xviii^	26.22 (5)	P3—O4—K1^xiv^	88.41 (12)
O4^xvi^—K1—P3^xviii^	103.21 (8)	Fe2—O4—K1^xiv^	117.90 (11)
O1^xi^—K1—P3^xvii^	79.13 (5)	K2^xiv^—O4—K1^xiv^	77.09 (8)
O1^xii^—K1—P3^xvii^	169.06 (5)	P3—O4—K2^xv^	82.65 (13)
O1^x^—K1—P3^xvii^	94.74 (5)	Fe2—O4—K2^xv^	87.54 (11)
O2^xvi^—K1—P3^xvii^	115.09 (6)	K2^xiv^—O4—K2^xv^	171.00 (11)
O2^xvii^—K1—P3^xvii^	26.25 (5)	K1^xiv^—O4—K2^xv^	94.06 (9)
O2^xviii^—K1—P3^xvii^	108.35 (6)		

Symmetry codes: (i) −*z*, *x*−1/2, −*y*+1/2; (ii) −*y*+1/2, −*z*, *x*−1/2; (iii) *x*−1/2, −*y*+1/2, −*z*; (iv) *z*, *x*, *y*; (v) *y*, *z*, *x*; (vi) *x*−1, *y*−1, *z*−1; (vii) −*x*+1, *y*−1/2, −*z*+1/2; (viii) *x*−1/2, −*y*+1/2, −*z*+1; (ix) −*x*+1/2, −*y*+1, *z*−1/2; (x) −*x*+1, *y*+1/2, −*z*+1/2; (xi) −*z*+1/2, −*x*+1, *y*+1/2; (xii) *y*+1/2, −*z*+1/2, −*x*+1; (xiii) −*x*+1, *y*−1/2, −*z*+3/2; (xiv) −*x*+3/2, −*y*+1, *z*−1/2; (xv) *x*−1/2, −*y*+3/2, −*z*+1; (xvi) −*y*+1, *z*+1/2, −*x*+3/2; (xvii) *z*+1/2, −*x*+3/2, −*y*+1; (xviii) −*x*+3/2, −*y*+1, *z*+1/2; (xix) −*y*+3/2, −*z*+1, *x*+1/2; (xx) −*z*+1, *x*+1/2, −*y*+3/2; (xxi) *x*+1/2, −*y*+3/2, −*z*+1; (xxii) *x*+1/2, −*y*+1/2, −*z*.
